# Infected Abdominal Aortic Aneurysm Successfully Treated With Endovascular Aortic Repair and Antibiotics: A Case Report

**DOI:** 10.7759/cureus.68287

**Published:** 2024-08-31

**Authors:** Tomohiro Nakajima, Tsuyoshi Shibata, Naomi Yasuda, Yutaka Iba, Nobuyoshi Kawaharada

**Affiliations:** 1 Cardiovascular Surgery, Sapporo Medical University, Sapporo, JPN

**Keywords:** bacteremia, fever, antibiotics, endovascular aortic repair, infected abdominal aortic aneurysm

## Abstract

Surgical treatment of infected aneurysms is problematic due to their high complication and mortality rates. Infected aortic aneurysms are at high risk of rupture and should be operated on as soon as possible after diagnosis. A 72-year-old female patient with a medical history of diabetes mellitus, hyperlipidemia, and hypertension presented with a fever of 38°C and back pain, without any apparent cause, in 2021. Her C-reactive protein (CRP) level increased to 20 mg/dL. Further evaluation with contrast-enhanced computed tomography (CT) revealed a low-density area with air pockets surrounding the abdominal aorta. The patient was diagnosed with native abdominal aortic infection and transferred to our hospital for treatment.

The next day, endovascular aortic repair (EVAR) was performed using an Endurant stent graft (161682). Postoperatively, the patient was treated with antibiotics, and subsequently, blood infection was alleviated. Moreover, the CRP levels normalized. Follow-up contrast-enhanced CT showed resolution of the air pockets surrounding the abdominal aorta. The patient was discharged home on postoperative day 33.

During her three-year follow-up as an outpatient, no recurrence of the infection was detected. While open surgical repair with prosthetic graft replacement is often the preferred treatment for infected abdominal aortic aneurysms, in select cases, as demonstrated by our patient, EVAR can be employed to prevent rupture, followed by antibiotic therapy to achieve infection control.

## Introduction

Native infected aortic aneurysms (IAAs) are associated with a high risk of rupture, and immediate surgical intervention is recommended upon diagnosis [1]. Surgical options typically include extraanatomical bypass grafting or in situ graft replacement. These procedures are often combined with an omental flap placement, if suitable. Several reports demonstrated graft replacement using rifampicin-soaked bioprosthetic grafts to enhance infection resistance [2]. In addition to prosthetic graft replacement and endovascular aortic repair (EVAR), other treatment options for infected abdominal aortic aneurysms (IAAAs) include aortic reconstruction using allografts or exclusion of the infected aneurysm following extra-anatomic bypass. EVAR is not recommended for IAAAs because the insertion of further prostheses into the infected area may prolong the infection.

In this case report, we present a patient with a suspected IAAA who underwent endovascular stent graft insertion to prevent vessel rupture followed by antibiotic therapy. The patient was discharged without requiring graft replacement surgery. We report our experience with this case, which showed no adverse events during the three-year follow-up period.

## Case presentation

A 72-year-old female with a medical history of urinary tract infection, hypertension, hyperlipidemia, diabetes mellitus, and no smoking presented with a fever of 38°C and back pain, without any apparent cause, in 2021. A week earlier, the patient had a fever of 38°C and persistent back pain. The patient was on a regimen of calcium channel blockers and statins; however, neither corticosteroids nor immunosuppressive agents were part of their medication regimen. Blood tests conducted in a local clinic revealed an elevated white blood cell count of 14,400 and a C-reactive protein (CRP) of 20.5 mg/dL (Table 1). Blood and urine cultures were also performed.

**Table 1:** Laboratory findings at the hospitalization. WBC: White blood cell

Test	Results	Units	Reference range
WBC count	14400	/μL	5000-9000
Hemoglobin	11.7	g/dL	14-18
Platelet count	307000	/μL	150000-450000
C-reactive protein	20.5	mg/dL	<0.1

**Figure 1:**
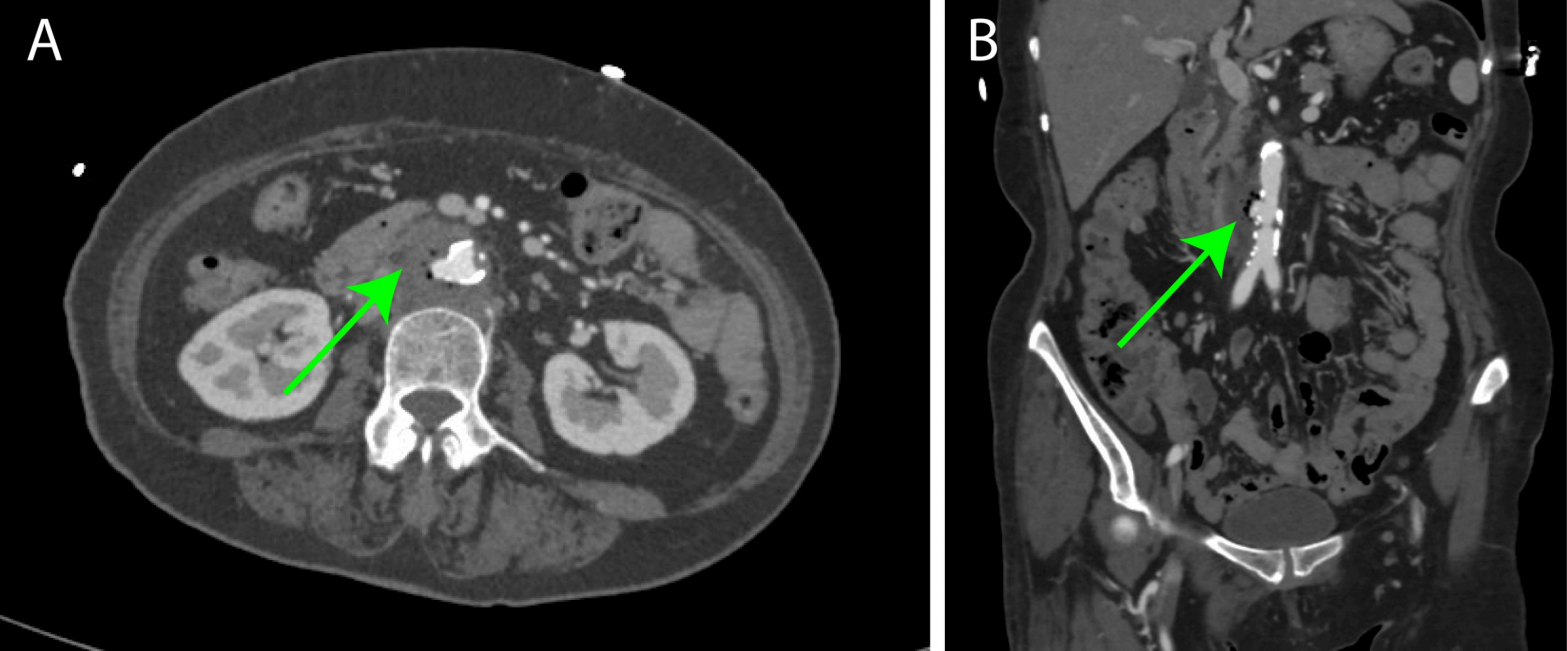
Preoperative enhanced computed tomography image (A) A contrast-enhanced CT scan revealed a penetrating atherosclerotic ulcer in a section of the abdominal aorta, with air pockets surrounding it (green arrow). (B) Coronal view.

Contrast-enhanced computed tomography (CT) revealed a low-density area with air pockets in a section of the infrarenal abdominal aorta (Figure 1). The diameter was 30mm. The proximal neck diameter was 14mm, and the length of the aneurysm, the diameter of access points, was 30mm. The patient was diagnosed with an IAAA, and empiric antibiotic therapy using vancomycin (2 g/day) and meropenem (3 g/day) was initiated.

**Figure 2:**
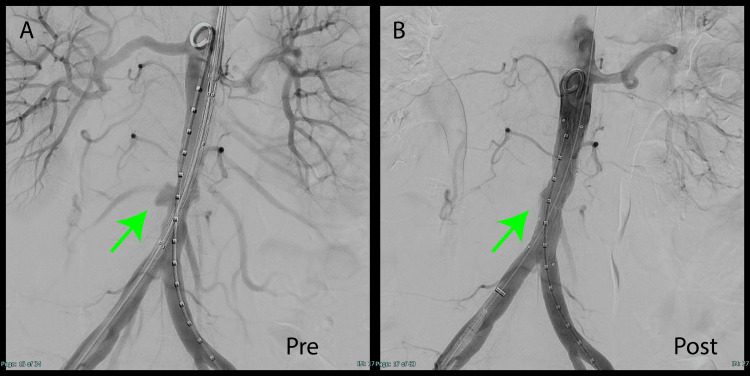
Intraoperative images (A) An abdominal angiography revealed a penetrating atherosclerotic ulcer in a section of the abdominal aorta (green arrow). (B) The follow-up imaging revealed a disappearance of contrast enhancement (green arrow).

**Figure 3:**
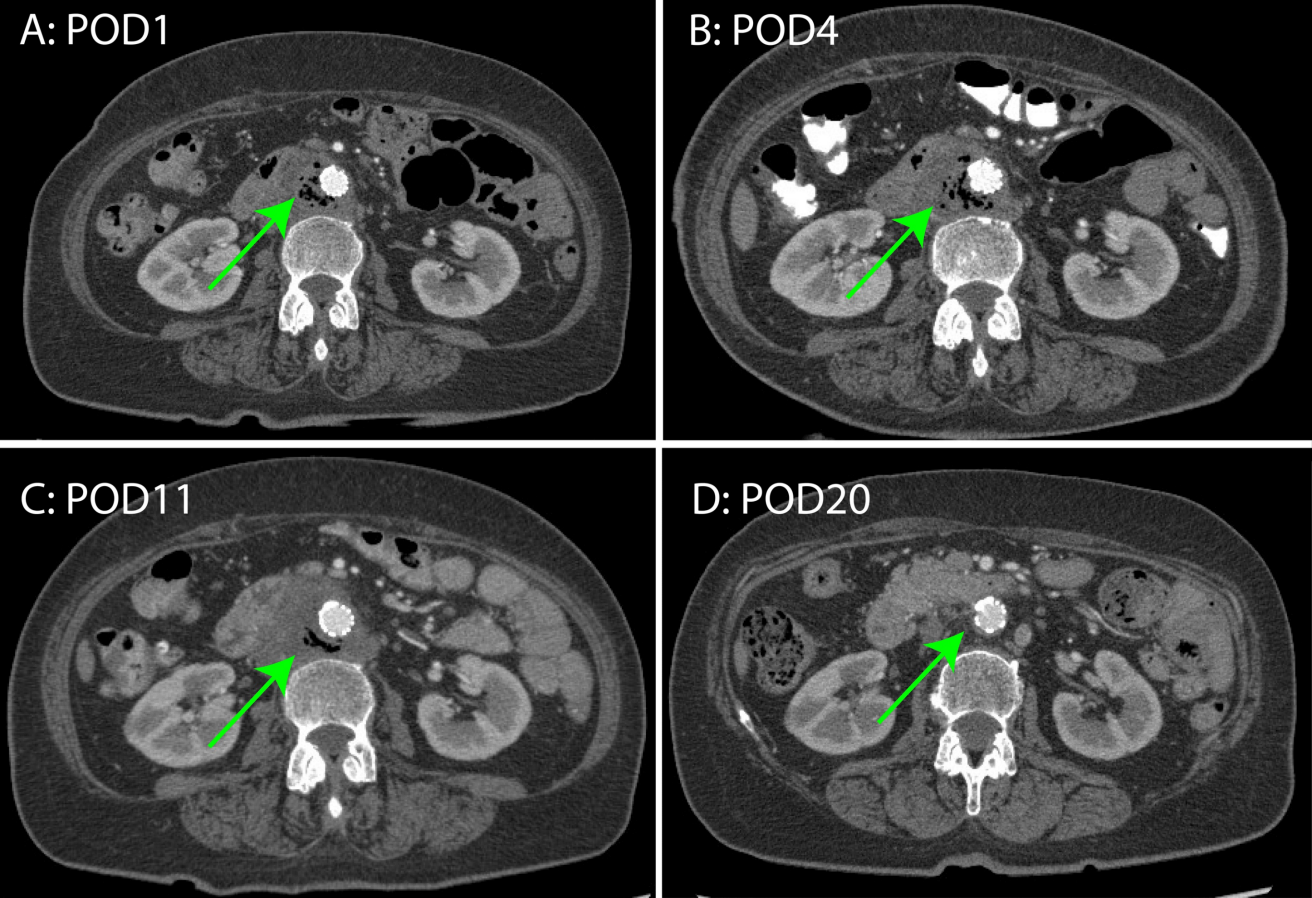
Postoperative enhanced computed tomography images (A) Postoperative day 1 (POD1). (B) POD 4. (C) POD 11. (D) POD 20. Over time, serial imaging studies revealed gradual resolution of periaortic air and complete dissipation of the inflammatory tissue surrounding the aorta (green arrows).

The next day, emergency EVAR was performed under general anesthesia using an Endurant stent graft (161682) to prevent rupture. Insertion of radifocus 150 cm and marker pig tail 5Fr from 8Fr sheath, guided to distal arch, guidewire changed to ultrastiff wire Insertion of pig tail catheter from 5Fr sheath, length of graft to be placed determined by contrast Endurant's leg. The 8Fr sheath was removed and the device was inserted through the right inguinal canal. The device was placed 10 mm peripherally from the renal artery as it was an infected aneurysm and there was a possibility of open surgery in the future (Figure 2). After EVAR, blood flow to the infected abdominal aortic protrusion was found to be absent. A contrast-enhanced CT performed on postoperative day 1 showed no blood flow into the aneurysmal sac (Figure 3A).

**Figure 4:**
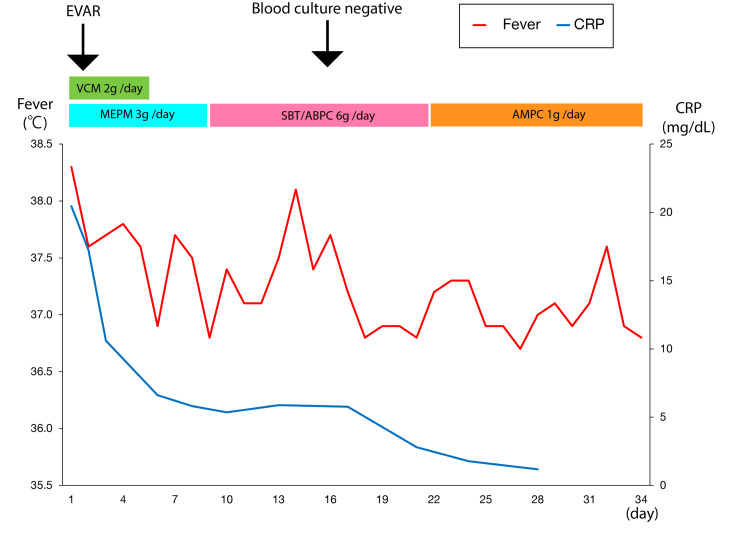
Chart showing the patient's clinical course during hospitalization EVAR: Endovascular aortic repair; CRP: C-reactive protein; VCM: vancomycin; MEPM: meropenem; SBT/ABPC: sulbactam/ampicillin; AMPC: amoxicillin.

On postoperative day 5, a blood culture revealed Proteus mirabilis infection. Antibiotic therapy was de-escalated to SBT/ABPC (6 g/day). The follow-up CT scans demonstrated a gradual absorption of periaortic air (Figure 3). Figure 4 illustrates the persistence of low-grade fever (around 37°C); however, CRP levels decreased.

On postoperative day 22, the antibiotic therapy was switched to oral AMPC therapy (1 g/day). The patient was discharged on postoperative day 33. She continued oral administration of AMPC (1 g/day) for three postoperative months, and subsequently, antibiotic therapy was discontinued.

**Figure 5:**
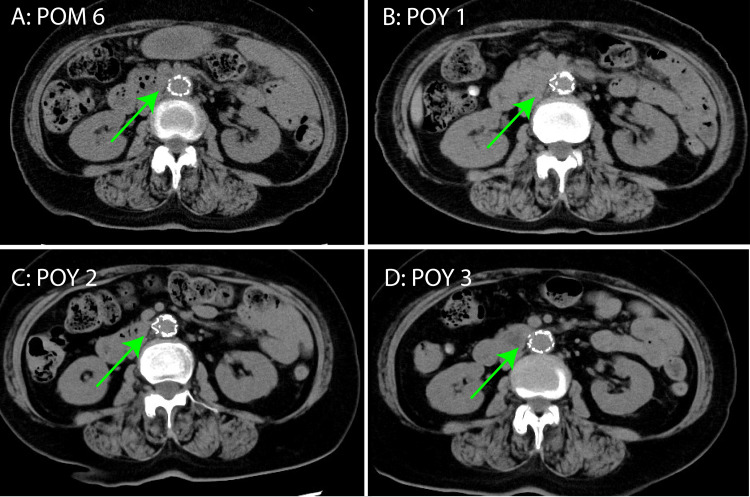
Postoperative enhanced computed tomography images (A) Postoperative month 6 (POM 6). (B) Postoperative year 1 (POY 1), (C) POY 2, and (D) POY 3. Periaortic air or signs suggestive of infection were not evident around the abdominal aorta (green arrow).

As shown in Figure 5, postoperative follow-up examinations at six months, one, two, and three years revealed no recurrence of the IAAA. The patient was monitored as an outpatient.

## Discussion

In this study, we report a rare case of an IAAA in a 72-year-old female who underwent an EVAR performed to prevent rupture. Native IAAs account for only 0.5-1.3% of all aortic aneurysms [3]. However, they exhibit a higher rupture rate than non-infected aneurysms, necessitating early diagnosis and treatment [4].

The key to management is identifying the causative organism, administering the appropriate antibiotic therapy, and excising the infected aneurysm. Traditionally, the approach has been to remove the infected tissue and perform an extra-anatomical bypass to avoid leaving foreign material in the infected area [5,6]. However, in-situ graft replacement with omental flap coverage is common. Some reports have suggested the effectiveness of rifampicin-soaked grafts in enhancing resistance to infections or the use of allografts; however, no consensus exists on these methods [7].

In this case, we initially performed EVAR to prevent rupture. We planned to perform this procedure using an open surgery for graft replacement and omental flap coverage at a later stage. However, as the inflammation gradually subsided, and the signs of the infected aneurysm disappeared, the patient was discharged. Follow-up over three years reflected no recurrence. Plain CT confirmed that there was no enlargement of the aortic aneurysm diameter and blood tests showed no recurrence of inflammation.

This patient benefited from early intervention and effective antibiotic therapy, ultimately avoiding open surgery. We speculate that some cases of IAAs can be successfully managed with less-invasive antibiotic therapy and EVAR, conducted to prevent rupture. However, the possibility of uncontrolled infection may necessitate the consideration of open surgical graft replacement as a potential next step in treatment.

There is no consensus regarding the duration of postoperative antibiotic therapy for infected aneurysms. Previous reports suggest that lifelong drug administration is preferred. In our case, antibiotic therapy was continued for three months after discharge.

This case highlights the potential for successful management of selected IAAs with EVAR and targeted antibiotic therapy, while emphasizing the need for close monitoring and readiness for more invasive interventions if necessary.

## Conclusions

We report the case of an IAAA in a 72-year-old female patient who was successfully treated with endovascular stent graft insertion, performed to prevent rupture, followed by antibiotic therapy; these treatments resolved the infection. The patient was followed up as an outpatient and no recurrence was reported for three years post-intervention.

Initially, open surgical debridement of the infected site was planned. However, the patient’s condition improved with antibiotic therapy. This case demonstrates the potential efficacy of a less-invasive approach combining endovascular intervention and targeted antibiotic therapy in managing select cases of IAAAs and highlights the significance of long-term follow-up to monitor for potential recurrence.
